# Effects of Human Respiratory Syncytial Virus, Metapneumovirus, Parainfluenza Virus 3 and Influenza Virus on CD4+ T Cell Activation by Dendritic Cells

**DOI:** 10.1371/journal.pone.0015017

**Published:** 2010-11-29

**Authors:** Cyril Le Nouën, Philippa Hillyer, Shirin Munir, Christine C. Winter, Thomas McCarty, Alexander Bukreyev, Peter L. Collins, Ronald L. Rabin, Ursula J. Buchholz

**Affiliations:** 1 Laboratory of Infectious Diseases, National Institute of Allergy and Infectious Diseases, National Institutes of Health, Bethesda, Maryland, United States of America; 2 Center for Biologics Evaluation and Research, U. S. Food and Drug Administration, Bethesda, Maryland, United States of America; University of Georgia, United States of America

## Abstract

**Background:**

Human respiratory syncytial virus (HRSV), and to a lesser extent human metapneumovirus (HMPV) and human parainfluenza virus type 3 (HPIV3), re-infect symptomatically throughout life without antigenic change, suggestive of incomplete immunity. One causative factor is thought to be viral interference with dendritic cell (DC)-mediated stimulation of CD4+ T cells.

**Methodology, Principal Findings:**

We infected human monocyte-derived DC with purified HRSV, HMPV, HPIV3, or influenza A virus (IAV) and compared their ability to induce activation and proliferation of autologous CD4+ T cells in vitro. IAV was included because symptomatic re-infection without antigenic change is less frequent, suggesting that immune protection is more complete and durable. We examined virus-specific memory responses and superantigen-induced responses by multiparameter flow cytometry. Live virus was more stimulatory than inactivated virus in inducing DC-mediated proliferation of virus-specific memory CD4+ T cells, suggesting a lack of strong suppression by live virus. There were trends of increasing proliferation in the order: HMPV<HRSV<HPIV3<IAV, and greater production of interferon-γ and tumor necrosis factor-α by proliferating cells in response to IAV, but differences were not significant. Exposure of DC to HRSV, HPIV3, or IAV reduced CD4+ T cell proliferation in response to secondary stimulus with superantigen, but the effect was transitory and greatest for IAV. T cell cytokine production was similar, with no evidence of Th2 or Th17 skewing.

**Conclusions, Significance:**

Understanding the basis for the ability of HRSV in particular to symptomatically re-infect without significant antigenic change is of considerable interest. The present results show that these common respiratory viruses are similar in their ability to induce DC to activate CD4+ T cells. Thus, the results do not support the common model in which viral suppression of CD4+ T cell activation and proliferation by HRSV, HMPV, and HPIV3 is a major factor in the difference in re-infectability compared to IAV.

## Introduction

Human respiratory syncytial virus (HRSV) is the most important viral agent of serious pediatric respiratory tract disease worldwide [1], [2], [3], [4], [5]. HRSV also can cause serious disease in the elderly and in immunosuppressed individuals [6]. Among the pediatric respiratory viruses, human parainfluenza virus type 3 (HPIV3) is the second most important cause of serious disease [7], [8], followed by human metapneumovirus (HMPV), which was first described in 2001 [9]. Similar to HRSV, HMPV is now recognized as an important agent of respiratory tract disease worldwide, especially in the pediatric and elderly populations [10], [11]. By contrast, the orthomyxovirus influenza A virus (IAV) causes serious disease in children and adults with the greatest burden of mortality being in the elderly [12].

These common human respiratory viral pathogens share a tropism for the superficial epithelial cells of the respiratory tract, although IAV appears to be much more cytopathic and also efficiently infects underlying cells in the epithelium [13], [14]. All four viruses cause acute infections with overlapping spectra of disease symptoms. Resolution of infection and protection against re-infection by these viruses are mediated by a similar array of immune mechanisms involving innate immunity, serum and secretory antibodies, and T cellular immunity. HRSV, however, is unusual (i) in its ability to efficiently infect and cause serious disease very early in life, with the peak of hospitalization at 2 months of age, and (ii) in its ability to readily re-infect throughout life without need of antigenic change. HMPV and HPIV3 also can cause serious disease in infancy, but not as early in life or as frequently as HRSV. HMPV and HPIV3 also can re-infect throughout life without need for antigenic change, but with less efficiency and severity than HRSV. In contrast, IAV differs from HRSV, HMPV, and HPIV3 in that infection usually induces long-lasting protection such that symptomatic re-infection is dependent on significant antigenic change. Finally, serious infection with HRSV during infancy is associated with more severe sequelae compared to these other viruses, including increased airway reactivity that can persist through childhood as well as possible links to asthma. These unusual features of HRSV epidemiology as compared to HMPV and HPIV3, in particular the greater frequency of infection and re-infection, are widely interpreted as evidence that HRSV has the ability to suppress or subvert the host adaptive immune response.

Conventional or myeloid dendritic cells (DC) are pivotal in initiating the adaptive immune response. Immature DC reside in peripheral tissue to capture antigen, and serve as sentinels to detect local infection, and in lymphatic tissue, where they encounter microbial macromolecules from the draining lymph. In addition, during a lower respiratory tract infection, the number of DC in bronchi and lung increases by chemotactic influx of precursors that originate primarily from circulating monocytes [15], [16], [17].

After pathogen recognition, the immature DC up-regulate major histocompatibility (MHC) and co-stimulatory molecules, express cytokines, and shift expression of chemokine receptors to direct the DC to the T lymphocyte-rich areas of lymphoid tissue. This process of DC maturation may be affected by additional cues from the infected tissue, such as cytokines produced by infected cells, immune cells, or dying cells. A major role of mature DC is to present antigen and activate CD4+ and CD8+ T cells such that they proliferate and are polarized into memory and/or effector subsets [18], [19], [20].

Naïve CD4+ T cells can differentiate into T helper (Th) subsets with distinct functions and effects on the adaptive immune response (reviewed in [21], [22], [23]). Four major CD4+ cell helper subsets are currently recognized, Th1, Th2, Th17, and follicular Th (T_FH_) [22], [24], [25]. Each of these subsets has signature cytokines. For example, for Th1 cells, the signature cytokine is interferon (IFN)-γ; for Th2 cells, it is IL-4; and for Th17 cells, it is IL-17A. Other cytokines may be expressed by each subset. For example, Th1 cells may also express Tumor necrosis factor (TNF)-α [26], [27]. Each of these subsets is to some extent self-stimulatory and reciprocally inhibitory [21], [23]. During re-infections, CD4+ T cell proliferation originates largely from antigen-specific memory CD4+ T cells that persist from the previous infection(s) and are re-activated by antigen-presenting DC [26], [28], [29].

Previously, we compared the effects of HRSV, HMPV, and HPIV3 on the maturation of human adult immature monocyte-derived DC in a side-by side *ex-vivo* study and found that each virus induced moderate, sub-maximal levels of MDDC maturation and cytokine/chemokine responses, without evidence of virus-specific impairment of MDDC maturation [30]. Although MDDC are not primary human DC, DC derived from primary human monocytes represent an appropriate model for lung DC, since monocytes give rise to myeloid DC in the resting lung [31] as well as to mucosal DC [32]. This is relevant to the immune response to HRSV that occurs in pulmonary mucosa [33]. MDDC generated using IL-4 and GMCSF treatment might not entirely match DC from the inflammatory environment of the lung, but they are phenotypically and functionally similar to DC located at sites of inflammation *in vivo* [34].

In the present study, we continue this analysis by measuring the ability of these viruses to induce proliferation and cytokine production by autologous CD4+ T cells. Because IAV induces stronger and more long lasting protection against symptomatic re-infection, we used this virus as a comparator to the three paramyxoviruses.

The T cell responses we investigated were those specific to the respective virus-derived antigens, which primarily represent stimulation of memory T cells from prior natural infection, and responses to the superantigen SEB, as a model of the effect of each virus on secondary T cell stimulation. In response to virus stimulation of MDDC, we found a strong virus-specific T cell proliferative response for each of the four viruses, with the response to IAV being somewhat (but not significantly) greater than that to HRSV and HMPV. In response to SEB secondary stimulation, T cell proliferation was transiently reduced in T cell cocultures with virus-matured MDDC, with the effect being greatest for IAV. Differences in the spectrum and quantity of cytokine production between the viruses were minimal. Thus, while these common respiratory viruses had an inhibitory effect on CD4+ T cell responses under certain conditions, the effect was not profound and unlikely to account for the ability of a virus to re-infect in nature since the effect was greatest for IAV.

## Results

### Comparison of the ability of human MDDC stimulated with rHRSV, rHMPV, rHPIV3, or IAV to induce proliferation of autologous CD4+ T cells

**Figure 1:**
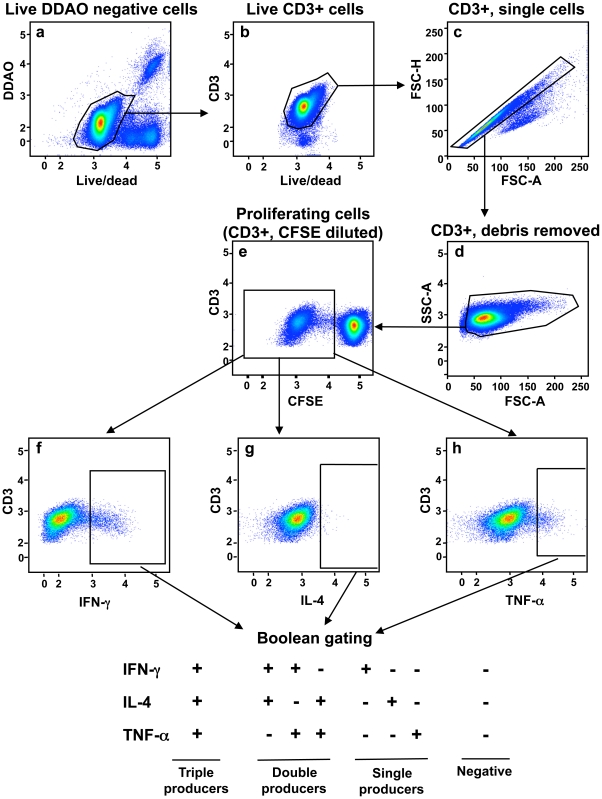
CD4+ T lymphocyte gating strategy. DDAO-labeled immature MDDC were exposed to virus or controls for 4–6 h, co-cultured for up to 1 week with autologous purified CSFE-labeled CD4+ T cells, and stimulated with PMA and ionomycin prior to staining for flow cytometry. Dead cells were excluded using a live/dead stain and MDDC were excluded based on the DDAO tracer (a). T cells were identified by gating on CD3+ cells (b). Single cells were identified using forward scatter height (FSC-H) and forward scatter area (FSC-A) to analyze cell size (c). Remaining debris was removed using FSC-A to analyze size and side scatter (SSC-A) to analyze cell complexity (d). Proliferating T cells were quantified by gating on CFSE to monitor the dilution of this tracer that occurs with cell division (e; proliferated cells are boxed). Live, proliferating, singlet CD3+ cells were analyzed for the expression of IFN-γ (f), IL-4 (g), and TNF-α (h). Boolean gating was used to quantify subsets listed at the bottom.

**Figure 2:**
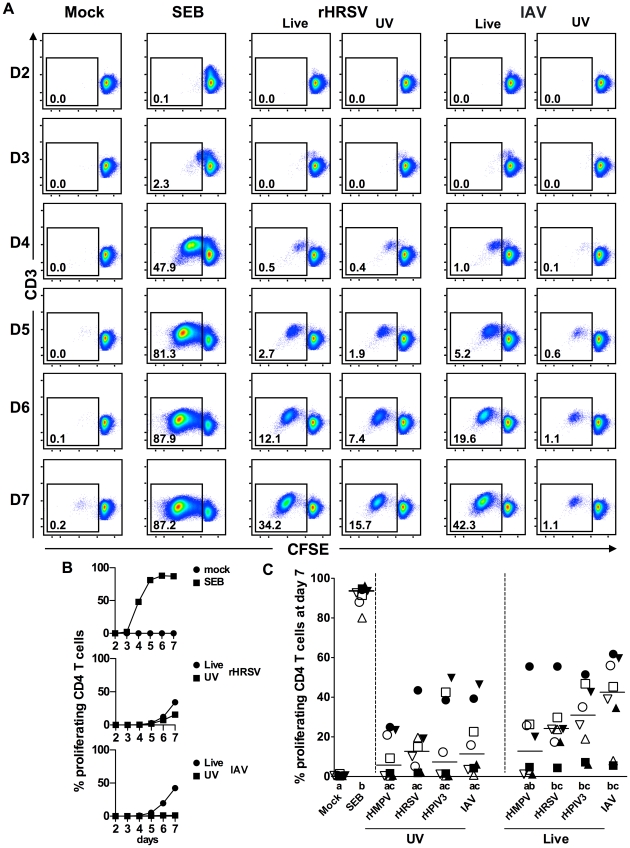
Proliferation of CD4+ T cells in response to autologous MDDC after exposure to SEB or purified virus. (A) Time course of CD4+ T cell proliferation in response to MDDC that were mock-treated or treated with SEB, rHRSV, or IAV, using cells from a single donor. Days of co-culture are indicated to the left. Proliferated cells are boxed, and their percentage relative to the total live population is indicated. (B) Line graphs of the data from panel A. (C) Summary of CD4+ T cell proliferation in experiments using cells from eight donors in which MDDC were mock-treated, or treated with each of the four live or UV-inactivated viruses or SEB, with proliferation measured following 7 days of co-culture. Each donor is represented by a unique symbol. The median percent proliferation for each condition is indicated by a horizontal line. Treatments sharing the same lowercase letter do not differ significantly at P≤0.05 (Friedman test with Dunns post hoc test (see Materials and Methods).

**Figure 3:**
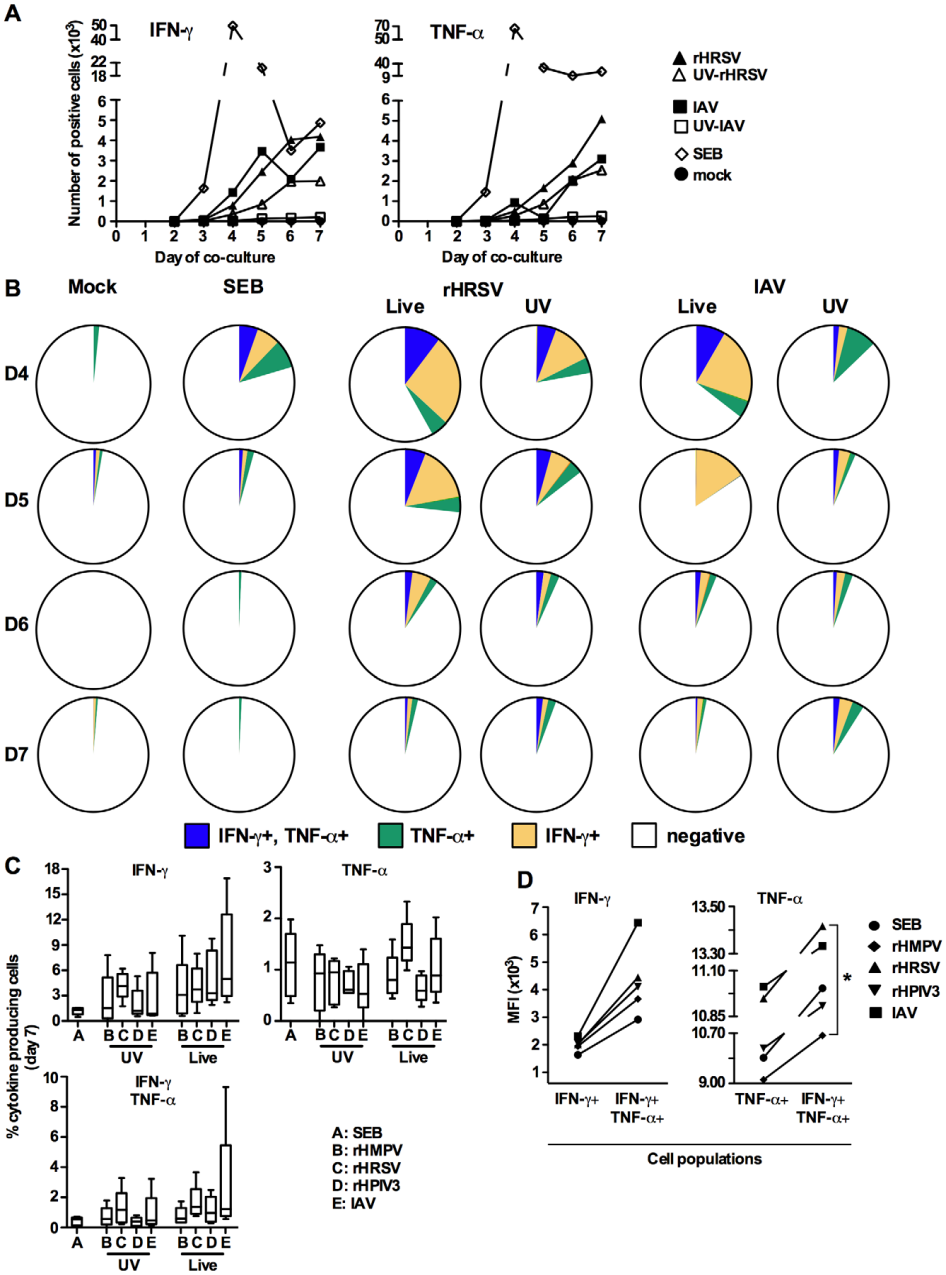
Cytokine expression by CD4+ T lymphocytes proliferating in response to MDDC treated with SEB or virus. This is a continuation of the experiment shown in Figure 2. Expression of IFN-γ, IL-4, and/or TNF-α individually and in each double or triple combination (Boolean gating) was determined in live, proliferating, singlet CD4+ T cells. (A, B) Time course of production of IFN-γ and/or TNF-α by proliferating CD4+ T cells proliferating in response to MDDC that were mock-treated or treated with SEB, or live or UV-inactivated rHRSV or IAV, using cells from the same donor shown in Figure 2 A and B. (A) Total number of proliferating CD4+ T cells expressing IFN-γ or TNF-α ~ (B) Percentages of proliferating CD4+ T cells expressing IFN-γ and/or TNF-α, shown as pie charts. (C) Expression of IFN-γ and/or TNF-α by live, proliferated CD4+ T cells in experiments representing five of the eight donors shown in Figure 2 C. Values are expressed as percentages of total proliferated CD4+ T cells. The box plots show the median (horizontal line) flanked by the 2^nd^ and 3^rd^ quartile. The outer bars show the range of values. No statistical differences were observed between the treatments. (D) Comparison of the MFI of IFN-γ (left panel) or TNF-α (right panel) by proliferating CD4+ T cells that were positive for only the single cytokine versus those positive for both cytokines. The data are from the experiment in part C (cells from five donors). Statistical differences are indicated by asterisks (Friedman test with Dunns post hoc test (see Materials and Methods); * = p≤0.05.

DDAO-labeled immature MDDC were mock-treated or treated for 4 to 6 h with rHMPV, rHRSV, rHPIV3, or IAV, an equivalent amount of each UV-inactivated virus, or, as a positive control, SEB. The MDDC were then washed extensively and co-cultured with autologous CFSE-labeled CD4 T lymphocytes at an MDDC-to-T cell ratio of 1∶10. After an incubation period of up to 7 days, the cells were stimulated with PMA and ionomycin and harvested, stained for discrimination between live and dead cells and immunostained for CD3 and several cytokines. CD4 T cells were analyzed by flow cytometry (see Figure 1 for the general scheme) to evaluate (i) proliferation, which was scored by dilution of CFSE dye that occurs with cell division (Figure 2), and (ii) cytokine production, measured by intracellular cytokine staining (Figure 3).

To determine the optimal time point for T cell analysis, we examined a time course of CD4+ T cell proliferation during co-culture with MDDC that had been exposed to mock-treatment, SEB, or a subset of the viruses, namely live or UV-inactivated rHRSV or IAV (Figure 2 A and B), using cells from one donor. CD4+ T cell proliferation was first detectable on day 2 in co-cultures with SEB-treated MDDC, and on days 4–5 with virus-stimulated MDDC, and continued to increase until day 6 or 7, respectively. As expected, mock treated MDDC induced a very low level of CD4+ T cell proliferation (∼0.2%), while SEB-treated MDDC induced the strongest proliferative response (87.6% at day 6). Among the virus specific responses, IAV-stimulated MDDC induced more CD4+ T cell proliferation (42.3% at day 7) than rHRSV-stimulated MDDC (34.2% at day 7), and UV-rHRSV- and UV-IAV-stimulated MDDC induced less proliferation (15.7 and 1.1% at day 7, respectively) than their live virus-stimulated MDDC counterparts. Proliferation was very low for cultures with UV-IAV-treated MDDC in this particular experiment, but cells from other donors proliferated in response to UV-inactivated virus to a greater extent (see Figure 2 C).

Using cells from one donor, we investigated whether the proliferating CD4+ T cells in the co-cultures with virus-stimulated MDDC were naïve or virus specific memory CD4+ T cells: this was determined on day 4, when proliferating cells were first evident, and also on day 7 (Figure S1). At day 4, for each virus, 92 to 95% of the cells expressed CD45RO and not CD45RA, showing that the first responding cells are virus-specific memory T cells. For each virus, about 50 to 60% of these CD45RO memory cells also expressed CCR7, a marker specific for central memory CD4+ T cells, whereas the remaining 40% to 50% did not express CCR7, which is typical for effector memory T cells. This indicates that the first proliferating cells in the co-cultures were memory rather than naïve cells, with a somewhat greater representation of central memory versus effector memory cells. On day 7, the proliferating CD4+ T cells were almost exclusively memory CD45RO+ cells, with a higher proportion of effector memory cells as compared to central memory cells (70% versus 30%).

Next, using cells from eight donors, we compared the level of CD4+ T cell proliferation in response to MDDC exposed to each of the four live or UV-inactivated viruses (rHMPV, rHRSV, rHPIV3, or IAV), or SEB, or mock-treatment (Figure 2 C). The cultures were assayed during the exponential phase of proliferation, on day 7, when the number of proliferating cells and the difference between live and UV-viruses was maximal. Again, mock-infected MDDC induced minimal levels of CD4+ T cell proliferation (median of 0.4% of proliferated cells), and SEB-treated MDDC induced maximal proliferation (median of 93.1%). With MDDC that had been stimulated with live virus, the percentage of divided CD4+ T cells increased in the order rHMPV < rHRSV < rHPIV3 < IAV (medians 12.4%, 23.8%, 30.6%, and 42.2%, respectively), although none of the differences among any of the groups was statistically significant. MDDC stimulated with IAV, rHPIV3, or rHRSV induced significantly more CD4+ T cell proliferation than mock-stimulated MDDC, whereas MDDC stimulated with rHMPV did not. MDDC treated with each of the four UV-inactivated viruses induced proliferation (median from 5.5% for UV-rHMPV to 12.6% for UV-rHRSV), but to a lower extent than the live-virus-treated MDDC counterparts, although the differences were not statistically significant. In separate experiments, using cells from four different donors, there was no significant CD4+ T cell proliferation during the first 24 h of incubation with MDDC stimulated with SEB or any of the live or UV-inactivated viruses or mock-treatment (not shown).

### Cytokine production by CD4+ T cells proliferating in response to MDDC stimulated with rHRSV, rHMPV, rHPIV3, or IAV

In addition to measuring proliferation with CFSE, we also measured CD4 T cell cytokine expression by blocking protein export with brefeldin A and staining for IFN-γ (Th1 cytokine), IL-4 (Th2 cytokine), IL-17 (Th17 cytokine), IL10 (immunosuppressive cytokine) and TNF-α (pro-inflammatory cytokine). In experiments employing cells from six donors, there were only minimal numbers of IL-4 or IL-17 producing cells at any time point (not shown) and a pilot experiment with cells from one donor, we detected only minimal numbers of IL10 producing cells. Therefore, we focused on the production of two Th1 cytokines IFN-γ and TNF-α.

From the same experiment shown in Figure 2 A and B, we analyzed the kinetics of cytokine production. In co-cultures with SEB-treated MDDC, the number of IFN-γ-positive CD4+ T cells increased from day 2 to day 4 and then decreased through day 7 (Figure 3 A). In co-cultures with MDDC stimulated with live or UV-inactivated IAV or HRSV, the number of IFN-γ-positive CD4+ T cells increased from day 3 to day 7, with the number of IFN-γ-producing cells being higher in response to live versus UV-inactivated virus (Figure 3 A). The response of TNF-α-positive cells followed a similar pattern. Figure 3 B shows a more detailed analysis of the proliferating CD4+ T cells that produced IFN-γ, and/or TNF-α, with cytokine expression presented as a percent of total proliferating cells. Figure 3 A and B show that, while the total number of cytokine-positive cells in cultures with virus-treated MDDC continued to increase through day 7 (Figure 3 A), the subset of the total proliferating cells that produced cytokines peaked on day 4 and decreased thereafter (Figure 3 B). Except on day 5, when a very low number of TNF-α+ cells were detected in the CD4+ population of this particular donor, co-cultures with rHRSV or IAV were quite similar with regard to the total number and the percentage of T cells expressing IFN-γ and/or TNF-α.

We then analyzed cytokine production on day 7 from five of the eight donors from the experiment shown in Figure 3 C. There were no significant differences between the viruses with regard to the extent of cytokine production or the Th1-biased nature of the memory CD4+ T cell response. The percentages of TNF-α positive and IFN-γ/TNF-α double-positive cells also were similar in response to MDDC treated with the various viruses. Also, the median fluorescence intensities (MFI) of T cells stained for IFN-γ and TNF-α were similar, showing that MDDC treated with each of the viruses were similar in their ability to induce IFN-γ and TNF-α production in proliferating CD4+ T cells (Figure 3 D).

Interestingly, the IFN-γ MFI was approximately two to three-fold greater in T cells that produced both IFN-γ and TNF-α, compared to cells that produced only IFN-γ. Similarly, TNF-α production was also higher in double positive cells than in single positive cells. In particular, IAV and rHRSV treated MDDC induced more TNF-α in double positive T cells than SEB, rHPIV3, and rHMPV (Figure 3 D). The finding that cells that express more than one cytokine have a higher level of expression has been noted previously for several other viral systems [35]. The percentage of cells expressing one or both cytokines and the intensity of expression are indicative of the quality of the memory CD4+ T cell response: in this regard, the responses induced by rHRSV, rHMPV, rHPIV3 were similar, whereas the response to IAV tended to be stronger.

### Effect of rHRSV, rHMPV, rHPIV3, and IAV treatment of MDDC on non-specific CD4+ T cell proliferation in response to SEB

It was previously reported that HRSV and HPIV3 suppress T cell responses to secondary stimuli [36], [37], [38], [39], [40]. We investigated whether this inhibitory effect is specific to HRSV and HPIV3, or is a more general property of these acute respiratory viruses. We used the superantigen SEB as the secondary stimulus, as also was done in previously published studies [36], [38], [41], [42]. Immature MDDC were mock-stimulated or stimulated for 4 to 6 h with rHMPV, rHRSV, rHPIV3, IAV or their UV-counterparts. The MDDC were then co-cultured with autologous CFSE-labeled CD4+ T lymphocytes in presence of SEB to drive non-specific T cell proliferation. The concentration of SEB was reduced from 1 µg/ml to 50 ng/ml, in an attempt to avoid overcoming possible viral effects.

**Figure 4:**
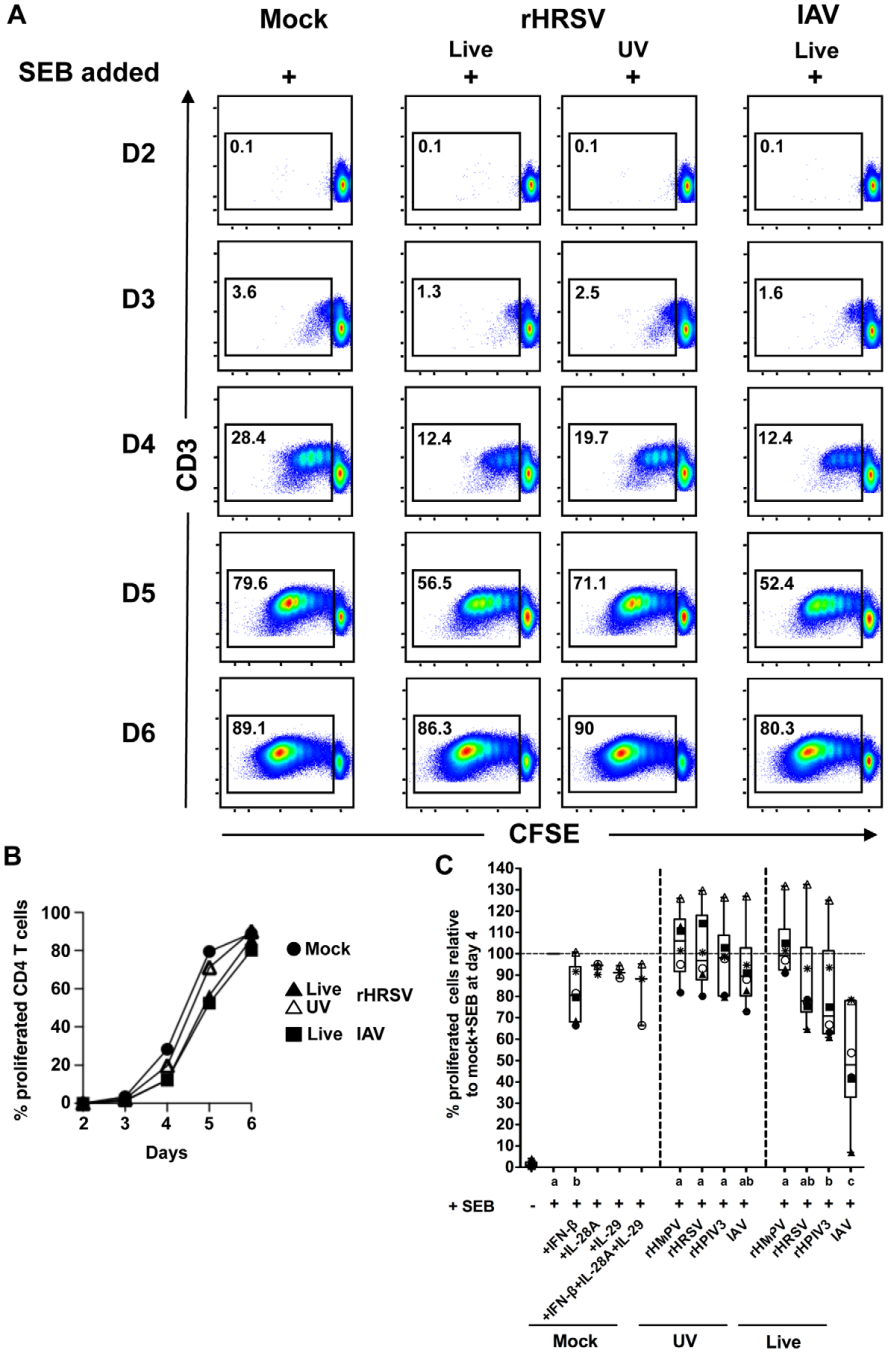
Proliferation of CD4+ T cells during co-culture with virus-treated MDDC and SEB. (A) Time course of CD4+ T cell proliferation in response to MDDC treated with rHRSV or IAV, or mock-treated, and co-cultured with SEB, using cells from a single donor. (B) Line graph of the data from panel A. (C) Summary of CD4+ T cell proliferation on day 4 in experiments representing cells from six donors, in which MDDC were mock-treated, or treated with each of the four live or inactivated virus, and co-cultured with SEB. Day 4 was used for comparison because the greatest differences between cultures containing mock, rHRSV, or IAV stimulated MDDC were observed on this day. For comparison, using cells from three of the six donors, mock-treated MDDC were co-cultured with autologous CD4+ T cells in the presence of SEB and IFN-β (75 IU per ml), or SEB and IL-28A (0,5 µg/ml), or SEB and IL-29 (0.5 µg/ml), or SEB and a cocktail of IFN-β, IL-28, and IL-29 (75 IU per ml, 0,5 µg/ml, 0,5 µg/ml, respectively). The box plots show the median (horizontal line) for each condition flanked by the 2^nd^ and 3^rd^ quartile. The outer bars show the range of values. Statistical differences are indicated by lower case letters (below the x axis). Treatment groups that share a letter are not significantly different.

As with the previous set of experiments, we first determined the best time point to measure proliferative and cytokine responses using cells from a single donor (Figure 4 A and B). As expected, SEB in combination with mock-treated MDDC resulted in a strong proliferation of CD4+ T cells (89.1% on day 6). Surprisingly, strong T cell proliferation also was observed by day 6 in response to MDDC that had been stimulated with virus prior to co-culture in the presence of SEB (86.3%, 90%, and 80.3% for rHRSV, UV-rHRSV, and IAV, respectively). Examination of earlier time points, however, indicated that there was a delay in T cell proliferation for the virus-stimulated cultures. Specifically, when compared to mock-pre-treatment, there was substantially less T cell proliferation on days 3, 4 and 5 in co-cultures with live rHRSV- and IAV-stimulated MDDC and, to a lesser extent, with UV-rHRSV-stimulated MDDC. Thus, exposure of MDDC to virus had an inhibitory effect on non-specific proliferation in response to SEB, but the inhibition of secondary proliferation by any of the respiratory viruses used for this study was transient, and, at least for this single donor, was not substantially different between the different viruses.

Next, using cells from six donors, we compared the level of CD4+ T cell proliferation in response to MDDC that had been mock-treated to those stimulated with each of the four live or UV-inactivated viruses (rHMPV, rHRSV, rHPIV3, or IAV) followed by co-culture in the presence of SEB (Figure 4 C). This comparison was made on day 4, the time point where prior virus treatment had the greatest inhibitory effect on CD4+ T cell proliferation in response to SEB. This showed that, compared to mock-treated MDDC, the proliferation was reduced by 22%, 29% and 52% by live rHRSV, rHPIV3 and IAV, respectively. T cells in co-cultures with rHMPV-stimulated MDDC proliferated in response to SEB to the same extent as those in co-cultures with mock-treated MDDC. Thus, based on the median of the responses of the six donors, rHMPV did not appear to have an inhibitory effect, whereas inhibition of T cell proliferation was slight for rHRSV and rHPIV3 and greatest with IAV. In contrast, and consistent with our previous report [36], none of the UV-inactivated viruses had significant anti-proliferative effects.

We previously reported that types I and III (lambda) IFN play a role in the inhibition of CD4+ T cell proliferation in response to HRSV-exposed MDDC [36]. Therefore, we asked whether adding IFN-β (which is the earliest type I IFN released from MDDC following stimulation by the whole panel of viruses [30]), IL-28A (IFN-λ2), or IL-29 (IFN-λ1), individually or combined, during co-culture might affect proliferation in response to SEB. Figure 4 C shows that among six donors, IFN-β (added at 75 IU/ml, a concentration representative of that detectable after exposure of MDDC to virus [30]) resulted in a significant 19% reduction of proliferation in response to SEB (P≤0.05 as compared to mock-treated MDDC). For three of the 6 donors, mock-treated MDDC were also co-cultured with CD4+ T cells in the presence of IL-28A or IL-29, or IL-28A, IL-29, and IFN-β together. We found that the type III interferons have limited, if any, additional suppressive effects.

### Effect of rHRSV, rHMPV, rHPIV3, or IAV treatment of MDDC on cytokine expression by CD4+ T cells proliferating in response to SEB

**Figure 5:**
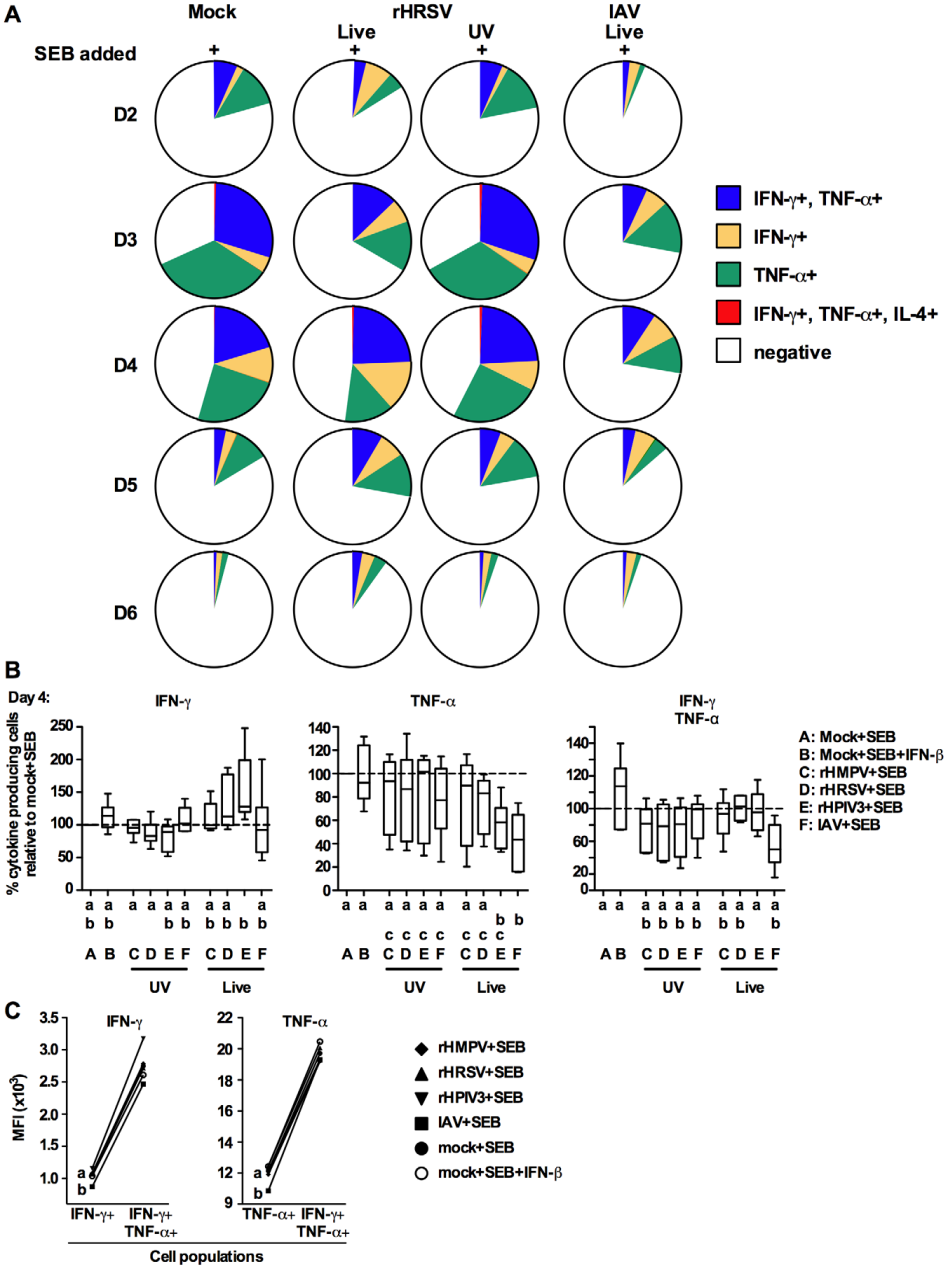
Cytokine expression by proliferating CD4+ T lymphocytes co-cultured with virus-treated MDDC and SEB. (A) Time course of cytokine production by CD4+ T cells proliferating in response to MDDC that had been mock-treated or treated with live or UV-inactivated rHRSV, or live IAV, and co-cultured in the presence of SEB, using cells from the same donor represented in Figure 4 A. The percentages of live proliferated cells positive for IFN-γ+, TNF-α+, IFN-γ+ plus TNF-α+ and IFN-γ+ plus TNF-α + plus IL-4+, are shown in the pie charts. (B) Percentage of live, proliferating CD4+ T cells positive for IFN-γ (left panel), TNF-α (middle panel), and IFN-γ plus TNF-α (right panel) after four days of co-culture with SEB and MDDC treated with each of the four live or UV-inactivated viruses or mock treatment, using cells from the same six donors shown in Figure 4 C. As a control, one co-culture for each donor containing mock-treated MDDC also contained 75 IU/ml IFN-β in addition to SEB. The box plots show the median (horizontal line) flanked by the 2^nd^ and 3^rd^ quartile. The outer bars show the range of values. (C) Comparison of the MFI of IFN-γ (left panel) or TNF-α (right panel) by proliferating CD4+ T cells that were positive for only the single cytokine versus those positive for both cytokines. The data are from the experiment in part B (cells from six donors). Treatments sharing the same lowercase letter do not differ significantly.

As part of the experiments described in the previous section, proliferating CD4+ T cells were analyzed by intracellular cytokine staining to quantify expression of IFN-γ, IL-4, IL-17 and TNF-α. Since there was minimal detection of cells producing IL-17 at any time point (data not shown), we focused on the production of IL-4, IFN-γ and TNF-α. However, IL-4+ cells that were detected were also IFN-γ+ and TNF-α+ (Figure 5A). The proportions of single IL-4 positive cells were very low and are not shown.

From the experiments shown in Figure 4 A and B, we analyzed the time course of cytokine production by proliferating CD4+ T cells, during co-culture in the presence of SEB, with MDDC that had been treated with live or UV-inactivated rHRSV, IAV, or mock treatment. In co-cultures of mock-treated MDDC and T cells with SEB, the fraction of proliferating T cells that expressed IFN-γ and/or TNF-α increased up to day 3 and then decreased thereafter (Figure 5 A). The time course of cytokine expression by proliferating T cells was nearly identical when co-cultured with UV-rHRSV-stimulated MDDC in presence of SEB. By contrast, live rHRSV modestly decreased the fraction of cytokine-producing cells on day 3, and modestly increased that fraction on day 5. IAV decreased the fraction of cytokine-producing cells on days 2–4. Thus, both live viruses effected a reduction in the fraction of cytokine-producing cells, especially on day 3, and the effect was greater for IAV than for rHRSV.

We then analyzed CD4+ T cell cytokine expression in the experiments shown in Figure 4 C, using cells from six donors and MDDC that were mock-stimulated or stimulated with each of the four live or UV-inactivated viruses (rHMPV, rHRSV, rHPIV3, or IAV) and then co-cultured in the presence of SEB (Figure 5 B and C). Cytokine expression by proliferating CD4+ T cells was assayed on day 4, the time point where prior virus treatment had the greatest inhibitory effect on CD4+ T cell proliferation in response to SEB (Figure 4 A). In general, the differences between the various treatments were minor. The median percentage of T cells expressing IFN-γ and/or TNF-α was similar for co-cultures containing MDDC that were initially mock-treated or stimulated with any of the UV-inactivated viruses. Thus, the response to the secondary SEB stimulation was not significantly affected by any of the UV-inactivated viruses. In the case of co-cultures with MDDC that were stimulated with live virus prior to the secondary SEB stimulation, the median percentages of cytokine-producing CD4+ T cells were not significantly different for rHRSV and rHMPV (and rHPIV3, except for TNF-α) versus mock-treatment (Figure 5 B). In the case of IAV, there was a significant reduction in the percentage of T cells expressing TNF-α, or both TNF-α and IFN-γ, as compared to mock. In addition, IAV reduced the IFN-γ and TNF-α MFIs in the IFN-γ+ and TNF-α + single positive population, respectively (Figure 5 C). Thus, overall, the response to secondary SEB stimulation was not significantly affected by prior infection with rHRSV, rHMPV, or rHPIV3, whereas there was a modest but significant inhibitory effect by IAV at day 4.

## Discussion

The ability of HRSV, HMPV and HPIV3 to re-infect symptomatically throughout life without the need for significant antigenic change has led to the widely held speculation that these viruses, especially HRSV, can suppress or subvert the host adaptive immune response, resulting in incomplete and inefficient long-term immunity. A number of studies have addressed virus-specific effects on APC and T lymphocyte responses *in vitro*, with varied and inconsistent conclusions. The first such studies reported that exposure of adult human peripheral blood mononuclear cells (PBMC) to HRSV, IAV, and Sendai virus suppressed proliferation in response to the non-specific mitogen phytohemagglutinin (PHA), an effect that was attributed to the expression of CD54/CD11a/CD18 (ICAM-1/LFA-1) and the interaction between APC and T cells [43], [44]. In 1992, Preston *et al.* [37] showed that exposure of human cord blood mononuclear cells to HRSV resulted in a reduction in proliferation in response to PHA. The same study showed that exposure of adult PBMC to HRSV reduced the proliferation response to Epstein-Barr virus antigen, although this effect was not seen with all of the tested HRSV strains. This effect was attributed to secreted IFN-α [45]. In another study, HPIV3 was shown to reduce proliferation of adult human PBMC in response to CD3-specific antibodies, an effect that was attributed to increased production of IL-10 [46].

More recent studies have used increasingly more defined conditions. Bartz *et al.* (2003) [41] generated human immature DC *in vitro* from cord blood CD34+ stem cells and showed that in presence of the toxic shock syndrome toxin-1 superantigen, HRSV caused increased DC apoptosis and reduced expression of IFN-γ and increased expression of IL-4 without affecting proliferation. Rothoeft *et al.* [38] reported reduced T cell proliferation in response to HRSV stimulated CD34-derived DC, as well as reduced IFN-γ expression. De Graaf *et al.* [42] showed that exposure of adult human MDDC to HRSV reduced SEB-mediated proliferation and cytokine production by naïve allogeneic CD4+ T cells, which was attributed to a soluble MDDC factor that was not identified but was not type I IFN. Chi *et al.* [36] also showed that exposure of adult MDDC to HRSV resulted in reduced proliferation of autologous CD4+ T cells in response to SEB or cytomegalovirus antigen, whereas HPIV3 and IAV controls resulted in less and no inhibition, respectively. The inhibitory effect of HRSV was partially mediated by type I and type III IFN [36]. Using human adult MDDC and allogeneic CD4+ T lymphocytes, Guerrero-Plata *et al.* [47] showed that HRSV inhibited the proliferation of allogeneic CD4+ T lymphocytes to a greater extent than HMPV. However, Jones *et al.* [48] did not observe HRSV-mediated inhibition of T cell proliferation under comparable conditions. Schlender et al. [39] showed that exposure to HRSV F protein expressed on the surface of epithelial cells inhibited proliferation of T lymphocytes in response to mitogen, but several of the co-culture studies described above provided evidence that ruled out contact inhibition in the observed inhibitory effects [36], [38], [42]. Thus, from these previous studies, there was inconsistency in the effects of HRSV on DC and on T cells and, in cases where inhibition or polarization was observed, there were differences in the proposed mechanism.

To address the question of virus-specific suppression of CD4 T cell function, we examined four viruses (HRSV, HMPV, HPIV3, and IAV) side-by-side, whereas the majority of the studies noted above examined a single virus, usually HRSV. While logistically more difficult, comparing a greater number of viruses provided for discrimination between effects that were unique to a particular virus versus those that were common to all. Also, analyzing more viruses and thus obtaining more comparisons for each individual donor was useful, given the heterogeneity of responses from an outbred human population. The “down-side” of our approach is that these studies were time consuming and laborious, and the donor-to-donor variability in human populations, and the relatively large panel of viruses studied, would have necessitated a high number of studies to reach statistical significance for the more nuanced differences between viruses, and forced us to interpret trends in differences.

We also used a more careful method of preparing virus. Viruses were grown in Vero cells, which do not produce type I IFN, and purified by sedimentation in sucrose gradients. We avoided the use of high input MOI of virus, especially with HRSV, which was used at an MOI of 10-20 or more in some studies [38], [41], [42], because HRSV is physically unstable, typically is contaminated with co-purified cellular membrane fragments, and has a high particle-to-PFU ratio [49]. Thus, the use of a high MOI could result in a large, disproportionate dose of viral antigen and cell contaminants. In contrast to a number of studies that used allogeneic (unmatched) cells, which results in T cell proliferation due to an MHC incompatibility that is not relevant to viral infection, we used autologous cells. Also, rather than relying on a single time point, we evaluated kinetics and magnitude of T cell proliferation and cytokine production. Another difference is that we (i) investigated responses specific to each of these viruses, which primarily represented stimulation of memory T cells from prior natural infection, and which were dependent on antigen processing and presentation by the DC, and (ii), using SEB as a model, addressed the effect of viral infection of DC on secondary antigenic responses.

With regard to antigen-specific responses, CD4+ T cell proliferation in response to MDDC exposed to rHRSV was less than that to rHPIV3 and IAV and greater than to rHMPV, but the differences were not significant. In addition, proliferation was greater in response to MDDC exposed to live versus UV-inactivated virus, indicating that, although we cannot rule out the possibility of viral interference with CD4+ T cell proliferation, the net effect of exposure to live virus was stimulatory rather than inhibitory. The increased percentage of proliferating cells found in IAV cultures might reflect the presence of more IAV-specific CD4+ T cells at the beginning of the culture compared to the frequency of T cells specific for the other viruses. However, the magnitude of T cell proliferation induced by the four viruses correlated well with the extent of MDDC maturation we observed previously [30], as well as in additional MDDC maturation studies (data not shown) in which IAV was slightly but not significantly stronger in inducing MDDC maturation than rHPIV3, rHMPV, and rHRSV. Thus, the trend of increasing T cell proliferation responses in the order: rHMPV < rHRSV < rHPIV3 < IAV might reflect the relative potency of each of these viruses to induce MDDC maturation [30]. The sub-maximal nature of MDDC maturation might represent insufficient stimulation rather than virus-mediated inhibition, since a secondary LPS stimulus further maturation to a similar final extent [30].

By day 7, the IFN-γ and TNF-α cytokine expression profiles were similar among the four viruses, with approximately the same proportion of IFN-γ single-positive or IFN-γ/TNF-α double-positive CD4+ T cells. This shows that MDDC stimulated by all four viruses induced the same cytokines at similar levels with no signs of inhibition or Th2- or Th17-biased responses.

The functionality of a protective T cell response depends on the quality of the cytokine producing cell. Several recent studies have shown that CD4+ T cells which are double-positive for IFN-γ and TNF-α produce these cytokines at a higher level compared to single-positive cells [26], [35], [50], [51]. This shows that these CD4+ T cells are more strongly activated, and more likely to provide stronger helper effects to CD8+ cells, leading to better protection [50], [51]. In the present study, we observed the same effect of increased cytokine production from double-positive cells as compared to single-positive cells. Thus, the proportion and number of IFN-γ/TNF-α positive cells was similar among the different viruses, providing no evidence of a deficit specific to any particular virus.

The CD4+ T cell recall response to all viruses was Th1-biased as characterized by the production of IFN-γ and the low IL-4 and IL-17 production by proliferating CD4+ T cells. This is offered with the caveat that the MDDC were generated *in vitro* in the presence of GM-CSF and IL-4, with the potential to bias the T cell response, in particular by stimulating or inhibiting the Th2 pathway. While there have been many reports of plasticity among CD4 T cell subsets, the data still points to a fair level of rigidity among Th1 and Th2 subsets, compared, for example, to Th17 and Treg subsets. This suggests that our *in vitro* observations reflect the level of Th1 responses to each of these viruses *in vivo*.

We also investigated whether any of the virus stimulated MDDC inhibited T cell proliferation and cytokine production to SEB, as a model of secondary infection that is independent of virus-mediated differential effects on antigen uptake and presentation pathways. Indeed, compared to their UV-inactivated counterparts, rHRSV, rHPIV3 and IAV inhibited the CD4 T cell response to SEB. However, this effect was transient, most pronounced on day 4, and showed little or no difference between live and UV-inactivated HRSV by day 6. The inhibitory effect was relatively less for live rHRSV and rHMPV, whereas live IAV and rHPIV3 induced a markedly stronger inhibition of proliferation and IFN-γ and TNF-α production by day 4 of co-culture.

The transient inhibition by live viruses in the SEB assay might be explained by the anti-proliferative effect of type I interferon on CD4+ T cells [52], [53]. We detected higher type I interferon expression levels in MDDC matured by rHPIV3 and influenza virus compared to the other viruses ([30], and data not shown), suggesting a possible role in inhibition of proliferation. We tested this hypothesis in the present study by treating the MDDC/T cell co-cultures with IFN-β in concentrations similar to those in supernatants of rHPIV3-exposed MDDC [30] and found a reduced proliferation in response to SEB, comparable to that of SEB-treated MDDC/T cell co-cultures with rHPIV3-MDDC. This supports the previous suggestion of Preston et al. [45] and Chi et al. [36] that type I IFN plays a role in inhibiting T cell responses to HRSV, and illustrates the importance of the local cytokine environment [20] in the modulation of memory T cell proliferation.

In summary, each of these common human respiratory pathogens can affect the ability of MDDC to activate CD4+ T cells. The more biologically relevant response, namely the proliferation of virus-specific memory T cells, was somewhat less for the paramyxoviruses compared to IAV. While this might make a contribution to a trend of increased ease of re-infection, the differences in proliferation did not rise to the level of statistical significance. The modestly reduced paramyxovirus-specific proliferative responses correlated with reduced levels of DC maturation observed in previous studies, an effect that appeared to reflect a lower level of stimulation rather than virus-mediated inhibition. There was no obvious virus-specific bias to T cell polarization, and cytokine production was not significantly different between viruses. The non-specific proliferation response to SEB was lower for IAV than for HRSV and the other viruses. These results suggest that rHRSV-infected MDDC do not strongly and specifically inhibit proliferation of CD4+ T cells. Thus, HRSV-specific effects on DC/T cell interactions are unlikely to account for the ability of HRSV to cause repeat infections during life.

## Materials and Methods

### Ethics statement

Elutriated monocytes and autologous CD4+ T lymphocytes were obtained from healthy donors at the Department of Transfusion Medicine of the National Institutes of Health, under a protocol (99-CC-0168) approved by the Institutional Review Board of the Clinical Center, NIH. Written informed consent was obtained from all donors.

### Virus stock preparation

Recombinant (r) HMPV (strain CAN97-83), rHRSV (strain A2) and rHPIV3 (strain JS) were described previously [13], [54], [55]. The rHMPV had previously been modified to silently remove tracts of A and T residues in the SH gene that had been sites of spontaneous mutations during passage *in vitro* [56]. Human IAV was the non-recombinant wild-type H3N2 A/Udorn/72.

Virus purification was described previously [30]. Briefly, Vero cells (CCL-81, ATCC, Manassas, VA) were infected with low-passage rHMPV, rHRSV, rHPIV3 or IAV at an input MOI of approximately 0.1 PFU/cell, and after 6 to 8 days, the supernatants were collected and clarified by centrifugation, and virus was collected by centrifugation on discontinuous sucrose gradients [30]. Since initial studies had indicated that the presence of sucrose interfered with MDDC maturation (results not shown), gradient purified viruses were pelleted by centrifugation to remove sucrose, as described [30]. Virus pellets were resuspended in Advanced RMPI (aRPMI) 1640 supplemented with 2 mM L-glutamine, and aliquots were snap frozen and stored at −80°C until use. Virus titers were determined by immunoplaque assay as described previously [54]. UV-inactivated viruses were included as controls and were prepared using a Stratalinker UV cross-linker (Agilent, Santa Clara, CA) at 0.5 J/cm^2^, with inactivation monitored by plaque assay.

### Generation of immature MDDC and purification of autologous CD4+ T lymphocytes

Elutriated monocytes and autologous CD4+ T lymphocytes were obtained from healthy donors at the Department of Transfusion Medicine of the National Institutes of Health, under a protocol (99-CC-0168) approved by the IRB of the Clinical Center, NIH. Written informed consent was obtained from all donors. As previously described [30], monocytes were subjected to CD14+ positive sorting on an AutoMACS separator (Miltenyi Biotec, Auburn CA), and were cultured in the presence of recombinant human IL-4 (R&D Systems, Minneapolis, MN) and recombinant human granulocyte-macrophage colony-stimulating factor (GM-CSF; Leukine®, Bayer Healthcare, Wayne, NJ) for 7 days to generate immature MDDC. Elutriated T lymphocytes were purified from contaminating red blood cells by centrifugation on Ficoll (Lymphocyte Separation Medium; Cellgro, Manassas, VA) followed by treatment with ACK lysis buffer (Lonza, Walkersville, MD) to lyse erythrocytes. T lymphocytes were frozen at −80°C. Prior to use, the cells were thawed, incubated overnight, and subjected to positive sorting on an AutoMACS separator using magnetic microbeads coated with a CD4 specific monoclonal antibody (Miltenyi Biotec) to obtain CD4+ T cells. The purity of the CD4+ T lymphocytes based on the cell surface expression of CD3 and CD4 proteins was confirmed by flow cytometry to be ≥96%.

### Treatment of MDDC with virus or controls

Immature MDDC were labeled with the far-red cell tracer 7-hydroxy-9H-(1,3-dichloro-9,9-dimethylacridin-2-one) (DDAO; Invitrogen, Frederick, MD). After 15 min incubation of MDDC in medium with 1.5 µM DDAO at room temperature, cells were quenched on ice using 5 volumes of aRPMI supplemented with 5% heat-inactivated human serum (Gemini Bio-Products, West Sacramento, CA) and extensively washed with aRPMI with 10% human serum. The DDAO labeled immature MDDC were then washed in aRPMI with 10% heat-inactivated fetal bovine serum (FBS), and seeded in 12-well plates at 6×10^5^ cells per well and were (i) keep mock-stimulated, (ii) infected for 4 to 6 h with live virus at an input MOI of 3 PFU/cell, (iii) with an equivalent amount of UV-inactivated virus, or (iv) incubated with 1 µg/ml of the superantigen SEB (Sigma, St Louis, MO), a strong inducer of CD4+ T cell proliferation. All inoculations or stimulations were performed in complete medium (aRPMI supplemented with 10% heat-inactivated FBS, 2 mM L-glutamine, 200 U/ml penicillin and 200 µg/ml streptomycin) at 37°C in 5% CO_2_.

### Co-culture of stimulated MDDC with autologous CD4+ T cells

After four to six h of stimulation, MDDC were extensively washed with complete medium and the absence of remaining infectious virus particles in the MDDC suspensions was confirmed by plaque assay as described above. To monitor CD4+ T cell proliferation by flow cytometry, autologous purified CD4+ T cells were labeled by incubation with the cell tracer carboxyfluorescein succinimidyl ester (CFSE, 1 µM) for 10 min at 37°C. Cells were quenched using medium with 5% human serum for 5 min on ice and extensively washed with complete medium. DDAO-labeled stimulated MDDC were co-cultured with CFSE-labeled CD4+ T lymphocytes at a ratio of one MDDC for ten CD4+ T lymphocytes for 1 to 7 days at 37°C in 5% CO_2_.

In some experiments (Figure 4 and 5), mock-treated MDDC, live viruses-treated MDDC or UV-viruses treated MDDC were co-cultured with autologous CD4+ T cells in the presence of SEB (50 ng/ml). In addition, mock-treated MDDC were co-cultured with autologous CD4+ T cells in the presence of (i) SEB (50 ng/ml), (ii) SEB plus IFN-β (75 IU/ml), (iii) SEB plus IL-28A (0.5 µg/ml), (iv) SEB plus IL-29 (0.5 µg/ml), or (v) SEB plus a cocktail of IFN-β, IL-28, and IL-29 (75 IU per ml, 0.5 µg/ml, 0.5 µg/ml, respectively).

### Flow cytometry

Co-cultures were incubated for 6 h at 37°C with 20 ng/ml Phorbol myristate acetate (PMA), 1 µM ionomycin and 10 µg/ml brefeldin A to prepare for intracellular cytokine staining. Cells were then harvested and stained using live/dead fixable blue dead cell stain (Invitrogen) for 30 min at 4°C to discriminate between live and dead cells by flow cytometry. The cells were fixed and permeabilized according to the manufacturers instructions using the perm/wash buffer kit (BD Biosciences, San Jose, CA), blocked with milk saponin (0.1% saponin, 0.1% bovine serum albumin, 5% non-fat dried milk in PBS) for 30 min and immunostained according to previously published protocols [57] with anti-human mAbs at concentrations previously determined to be optimal by titration. The following anti human monoclonal antibodies were obtained from BD biosciences unless otherwise stated: CD3 allophycocyanin-cychrome 7 (APCcy7), clone SK7; IFN-γ phycoerythrin-cychrome 7 (PECy7), clone 4S.B3; IL-4 phycoerythrin (PE), clone MP4-25D2; TNF-α biotin, (clone Mab11) followed by QDot605-streptavidin (Invitrogen); and in some experiments (not shown), IL10 (APC) clone JES3-19F1, IL-17A fluorescein isothiocyanate (FITC) clone eBio64DEC17 (eBioscience, San Diego, CA). The steps in the flow cytometry analysis are shown in Figure 1. At least 30,000 events were acquired using a BD LSRII flow cytometer (BD Biosciences).

In addition, in some experiments, we determined if the proliferating CD4+ T cells arose from naïve versus virus specific memory cells. Virus stimulated MDDC were co-cultivated with CFSE stained autologous CD4+ cells. At day 4 and 7, cells were harvested and stained with live/dead fixable violet dead cell stain (Invitrogen) to discriminate between live and dead cells, and with an antibody specific to CD3 (APCcy7, clone SK7) to identify T lymphocytes. To discriminate between naïve and memory CD4+ T cells, cells were stained with antibodies to CD45RA (PE, clone HI100), and to CD45RO (PEcy5, clone UCHL1), respectively. To discriminate between central memory and effector memory CD4+ T cells, co-cultures were stained for CCR7 (PEcy7, clone 2H4). CCR7 is expressed on central memory but not on effector memory CD4+ T cells [58].

Compensation was performed automatically using single color antibody capture beads (BD biosciences) for each antibody. Due to cell number limitations, settings and gating were adjusted using fluorescence minus one controls in five independent experiments with the same staining panel of antibodies and cell numbers as used in the present study. The gating was performed generously (i.e. far enough from the negative populations to not include negative events in the positive gates). The gating for cytokines was kept consistent between experiments as rainbow beads were used to adjust each photomultiplier tube voltage to the same median fluorescence for all experiments as previously described [35], [59], [60]. In addition, fluorescent minus one controls were included in one experiment presented in the present study. In each experiment and each fluorescence minus one control, no cells negative for a given cytokine were ever found in the positive gate. Live/dead staining, forward scatter height, forward scatter area, side scatter, DDAO, CD3, CFSE, and expression of IFN-γ, IL-4, IL-17 and TNF-α were analyzed using FlowJo version 8.8.2 software (©Tree Star, Inc., Ashland, OR). Pie charts were generated using SPICE version 4.2.3, a data mining and visualization software for multicolor flow cytometry, written and kindly provided by Mario Roederer and Joshua Nozzi of the Vaccine Research Center at the National Institute of Allergy and Infectious Diseases.

### Statistical analysis

Data sets were assessed for significance using parametric one-way repeated measures ANOVA with the Tukey post hoc tests for normally distributed data sets or the non-parametric Friedman test with Dunns post hoc test. A log_10_ transformation was applied to data sets when necessary to obtain equal standard deviation among groups, a necessary requirement of both tests. Statistics were performed using Prism, version 5 (© 1992-2008 GraphPad Software, Inc, San Diego, CA). Data were only considered significant at P≤0.05.

## Supporting Information

Figure S1 **Memory phenotype of the proliferating CD4+ T cells.** Proliferating CD4+ T cells (CFSE diluted T cells) were analyzed for naïve or memory markers. MDDC derived from one donor were stimulated with the indicated virus at an MOI of 3 and co-cultivated with autologous CD4+ T cells at the ratio of 1 MDDC for 10 CD4+ T cells. The phenotype of the proliferating CD4+ T cells was evaluated on day 4, corresponding to the time of detection of the first proliferating cells (see Figure 2 A), and on day 7, corresponding to the time point when proliferation and cytokine production was evaluated for multiple donors (see Figure 2 C, and Figure 3 C and D). The percentage of live proliferated cells positive for CD45RA, CD45RO and CCR7 are indicated.
(TIF)
