# Supplementary figures and images for: Effects of Human Respiratory Syncytial Virus, Metapneumovirus, Parainfluenza Virus 3 and Influenza Virus on CD4+ T Cell Activation by Dendritic Cells
Source: PLoS One. 2010 Nov 29;5(11):e15017. doi: 10.1371/journal.pone.0015017 (PMC2993941; doi:10.1371/journal.pone.0015017)

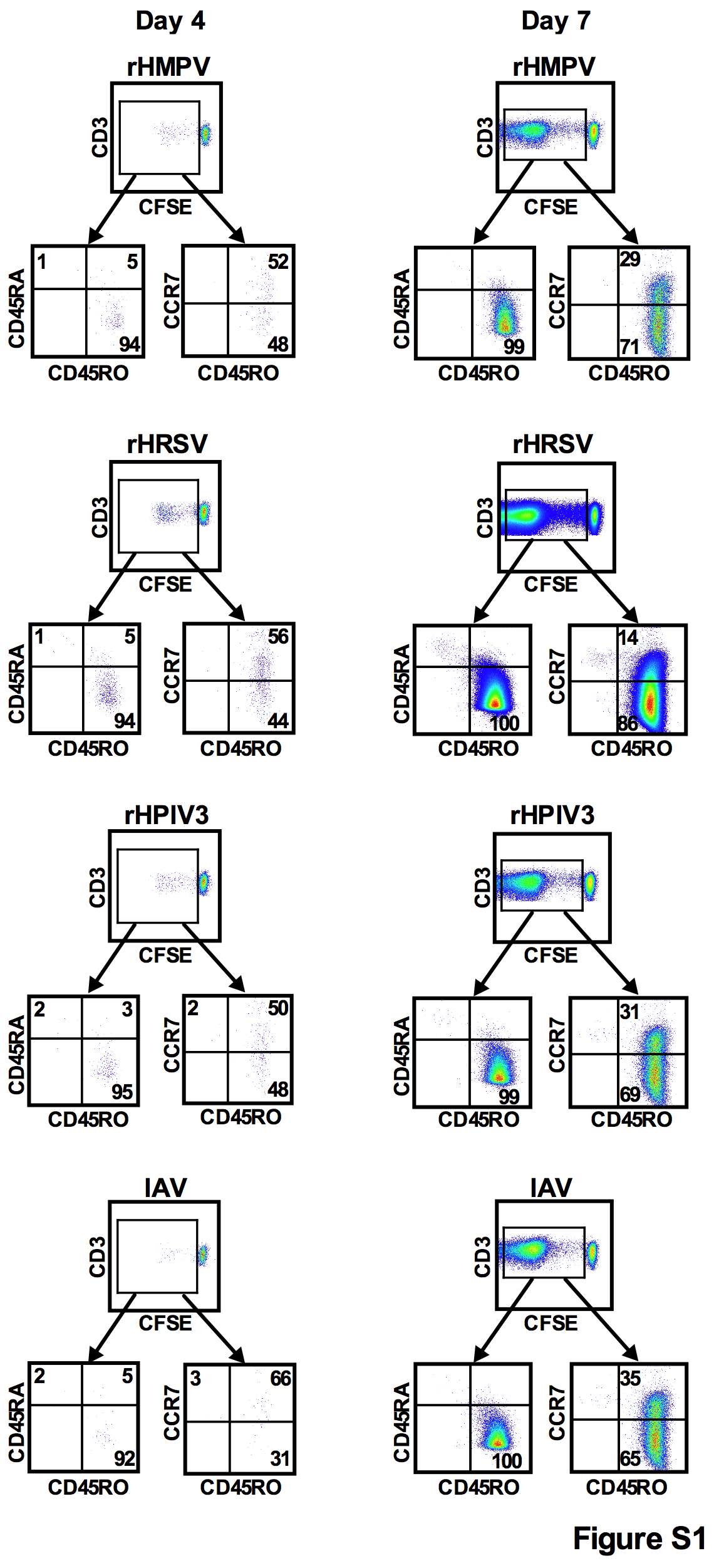

Supplement: Figure S1 — Memory phenotype of the proliferating CD4+ T cells. Proliferating CD4+ T cells (CFSE diluted T cells) were analyzed for naïve or memory markers. MDDC derived from one donor were stimulated with the indicated virus at an MOI of 3 and co-cultivated with autologous CD4+ T cells at the ratio of 1 MDDC for 10 CD4+ T cells. The phenotype of the proliferating CD4+ T cells was evaluated on day 4, corresponding to the time of detection of the first proliferating cells (see Figure 2 A), and on day 7, corresponding to the time point when proliferation and cytokine production was evaluated for multiple donors (see Figure 2 C, and Figure 3 C and D). The percentage of live proliferated cells positive for CD45RA, CD45RO and CCR7 are indicated. (TIF) [file pone.0015017.s001.tif]
